# Supplementary material for: A comparative analysis of APGAR score and the gold standard in the diagnosis of birth asphyxia at a tertiary health facility in Kenya
Source: PLoS One. 2023 May 24;18(5):e0285828. doi: 10.1371/journal.pone.0285828 (PMC10208496; doi:10.1371/journal.pone.0285828)
Supplement: S1 File — (DOCX) [file pone.0285828.s001.docx]

# Supplementary Material 1: Data Collection Form (Maternal and Neonatal)

1. **DATA COLLECTION FORM FOR NEONATES**

SECTION A: SOCIO-DEMOGRAPHIC INFORMATION FOR MOTHERS OF NEONATES

| 1 | ID number | (Participant number/day/month of year) |
| --- | --- | --- |
| 2 | Residence |  |
| 3 | Gestational age  -Gestational age by LMP  -Gestational age by Ballard’s score | (in completed weeks)  (in completed weeks) |

SECTION B: LABOUR PROCESS

| 4 | Referred or not | 0 = Yes, 1 = No |
| --- | --- | --- |
| 5 | Length of labour (in number of completed hours) |  |
| 6 | Induction of labour | 0 = Yes, 1 = No |
| 7 | If Yes, what was the indication? |  |
| 8 | Mode of delivery | 0=vaginal delivery 1=instrumental vaginal delivery 2=caesarean section |
| 9 | What was the indication (for 1 and 2 only) |  |
| 10 | Time of delivery | 0= morning, 1=afternoon, 2=evening, 3=night |

SECTION C: ASSESSMENT OF THE NEONATE

| 11 | APGAR score (1^st^, 5^th^ and 10^th^ minute) | 1^st^ minute:  A =  P =  G =  A =  R =  Total = |
| --- | --- | --- |
|  |  | 5^th^ minute:  A =  P =  G =  A =  R =  Total = |
|  |  | 10^th^ minute:  A =  P =  G =  A =  R =  Total = |
| 12a | Who scored the baby? | 0=Student 1=Midwife 2=Paediatric resident 3=Paediatric consultant |
| 12b | Sub-cadre of midwife | 0=diploma, 1=BSc, 2=MSc, 3=Others |
| 13 | Umbilical cord pH at birth |  |
| 14 | Umbilical cord pH in the fifth minute |  |
| 15 | Sex | 0=male, 1=female |
| 16 | Weight (In g) |  |
| 17 | Head circumference (in cm) |  |
| 18 | Presence of seizures | 1 = Yes, 0 = No |
| 19 | If yes to 18 above | 0 = at birth  1 = <6hrs  2 = 6-12hrs  3 = 12-24hrs |
| 20 | Presence of altered tone | 1 Yes, 0 = No |
| 21 | If yes to 20 above | 0 = at birth  1 = <6hrs  2 = 6-12hrs  3 = 12-24hrs |
