# Supplementary material for: A comparative analysis of APGAR score and the gold standard in the diagnosis of birth asphyxia at a tertiary health facility in Kenya
Source: PLoS One. 2023 May 24;18(5):e0285828. doi: 10.1371/journal.pone.0285828 (PMC10208496; doi:10.1371/journal.pone.0285828)
Supplement: S2 File — (DOCX) [file pone.0285828.s002.docx]

# **Supplementary Material 2: Data Collection Form (Healthcare Workers)**

1. **DATA COLLECTION FORM FOR THE HEALTHCARE PROVIDERS**

SECTION A: SOCIO-DEMOGRAPHIC INFORMATION

| 1 | ID number | (Participant number/day/month of year) |
| --- | --- | --- |
| 2 | Age | (In years) |
| 3 | Sex | 0=male, 1=female |
| 4a | Cadre | 0=Student 1=Midwife 2=Paediatric resident 3=Paediatric consultant |
| 4b | Sub-cadres for midwives | 0=Diploma, 1=Bsc, 2=Msc, 3=others, specify |
| 5 | Number of years of working experience | 0= <5years, 1= 5-10years, 2= 10-15years  3= >15years |

SECTION B: FACTORS INFLUENCING APGAR SCORING

| 6 | Do you give APGAR scores taking into account the individual parameters A, P, G, A, R? | 0 = Yes, 1 = No |
| --- | --- | --- |
| 7 | Do you have ready access to the APGAR scoring chart? | 0 = Yes, 1 = No |
| 8 | Do you feel the need to refer to the APGAR scoring chart before giving a score to a baby? | 0 = Yes, 1 = No |
| 9 | If yes to 8 above, how often? | 0 = seldom (once a month)  1 = sometimes (<5 times a month)  2= often times (5-10times a month)  3= frequently (more than 10 times a month)  4= all the time |
| 10 | Have you ever needed a second opinion to give a baby’s APGAR score? | 0 = Yes, 1 = No |
| 12 | Do you document APGAR scores immediately you assign them in the first, fifth and tenth minutes? | 0 = Yes, 1 = No |
| 13 | Do you consider any of the following when providing an APGAR score? Tick all that apply   - A colleague’s opinion of what the score should be - The need for resuscitation - The nature of resuscitation you do - How long you resuscitate - The time of the day you carry out the delivery - The type of delivery (SVD or CS) |  |
