# Supplementary material for: A comparative analysis of APGAR score and the gold standard in the diagnosis of birth asphyxia at a tertiary health facility in Kenya
Source: PLoS One. 2023 May 24;18(5):e0285828. doi: 10.1371/journal.pone.0285828 (PMC10208496; doi:10.1371/journal.pone.0285828)
Supplement: S3 File — (DOCX) [file pone.0285828.s003.docx]

**Supplementary Material 3: STANDARD OPERATING PROCEDURE FOR UMBILICAL CORD BLOOD COLLECTION**

Standard operating procedure (SOP) for umbilical cord blood collection is adapted from the 2015 guidelines of the Research Centre for Women’s and Infants’ Health (RCWIH) BioBank in Canada for umbilical cord blood collection (RCWIH BioBank, 2015).

- We would prepare a heparinized syringe by taking 0.1ml of Galaxy’s heparin (heparin sodium 5000IU/ml) and putting into a sterile 2cc Revital® syringe fitted with its 23G needle and keep.
- Once the baby is born, we would double-clamp the umbilical cord blood immediately after delivery. One clamp will be placed towards the placental end and the other clamp towards the neonatal end.
- The placenta will be placed such that its fetal surface and the umbilical cord are facing up. Any cord knots (true or false) or entanglements will be left in the manner received and not untangled.
- Umbilical cord blood will then be collected by carefully inserting the pre-heparinized syringe above the clamp site at a suitable angle that enables penetration of one vessel wall but not both walls. To prevent personal injury, we would avoid placing our hands immediately behind the intended site of penetration when collecting the blood.
- Once the needle tip is secured within the vessel, we would pull the syringe plunger gently to draw out the cord blood. We would then gently move the needle tip within the vessel to maintain blood collection while pulling the plunger, if the need arises.
- Once we obtain our sample, we would clamp the cord above the site of venipuncture.
- We would carefully dispose of the needle with the syringe in a biohazardous sharps container.
- We would then analyse the collected sample in the SIEMENS RAPIDPoint® BGA analyser.
