# Supplementary material for: A comparative analysis of APGAR score and the gold standard in the diagnosis of birth asphyxia at a tertiary health facility in Kenya
Source: PLoS One. 2023 May 24;18(5):e0285828. doi: 10.1371/journal.pone.0285828 (PMC10208496; doi:10.1371/journal.pone.0285828)
Supplement: Attachment — Submitted filename: MAN ADEBOYE EDITED PONE-D-22-21860.pdf [file pone.0285828.s005.pdf]

PLOS ONE

Assessment of effective use of APGAR score in comparison to the gold standard in diagnosis of birth asphyxia at Moi Teaching and Referral Hospital, Kenya.

--Manuscript Draft--

**Manuscript Number:**	PONE-D-22-21860
**Article Type:**	Research Article
**Full Title:**	Assessment of effective use of APGAR score in comparison to the gold standard in diagnosis of birth asphyxia at Moi Teaching and Referral Hospital, Kenya.
**Short Title:**	Effectiveness of APGAR score compared to Gold Standard compared to Gold Standard among newborns
**Corresponding Author:**	Albertine Enjema Njie, MD<br>Moi University School of Medicine<br>Eldoret, KENYA
**Keywords:**	APGAR Score, umbilical cord pH, Birth Asphyxia, Western Kenya.
**Abstract:**	Background : Birth asphyxia is a consistent key contributor to neonatal morbidity and mortality, notably in sub-Saharan Africa. The APGAR score is a globally used diagnostic tool for birth asphyxia; but there is paucity of data comparing this score with the gold standard (umbilical cord blood pH <7 with neurologic involvement), as well as limited studies assessing how effectively healthcare providers apply the score.<br>Objective: This study determined how effectively the APGAR score is used in Moi Teaching and Referral Hospital (MTRH) compared to the gold standard in diagnosis of birth asphyxia and identified the healthcare provider factors that affect the effective use of the score.<br>Methods : Using a quantitative cross-sectional hospital-based design, term babies born in MTRH who weighed ≥2500g were randomly and systematically sampled; and healthcare providers who assign APGAR scores were enrolled via a census. Umbilical cord blood was drawn at birth and at 5minutes for pH analysis. APGAR scores assigned by healthcare providers were recorded. Effective use of the APGAR score was determined by sensitivity, specificity, positive and negative predictive values. At a significance level of 0.05, multiple logistic regression analysis identified independent healthcare provider factors associated with effective use of the APGAR score.<br>Results: We enrolled 102 babies, and 50 (49%) were females. Among the 64 healthcare providers recruited, 40 (63%) were female and the median age was 34.5years [IQR: 31.0, 37.0]. Assigned APGAR scores had a sensitivity of 71% and specificity of 89%. Babies who were resuscitated, those born via instrumental delivery, and those scored by healthcare providers without access to APGAR scoring charts were significantly more likely to get incorrect APGAR scores.<br>Conclusion: Assigned APGAR scores had low sensitivity and positive predictive values. Healthcare provider factors independently associated with effective APGAR scoring include instrumental delivery, lack of access to APGAR scoring charts, and neonatal resuscitation.
**Order of Authors:**	Albertine Enjema Njie, MD
	Winstone Mokaya Nyandiko, MBchB, MMed, MPH
	Phinehas Ademi Ahoya, MBchB, MMed
	Jude Suh Moutchia, BSc, MSc, PhD
**Additional Information:**	
**Question**	**Response**
**Financial Disclosure**<br><br>Enter a financial disclosure statement that describes the sources of funding for the work included in this submission. Review	The author(s) received no specific funding for this work.

Powered by Editorial Manager® and ProduXion Manager® from Aries Systems Corporation

the submission guidelines for detailed requirements. View published research articles from *PLOS ONE* for specific examples.<br><br>This statement is required for submission and **will appear in the published article** if the submission is accepted. Please make sure it is accurate.<br><br>**Unfunded studies**<br>Enter: *The author(s) received no specific funding for this work.*<br><br>**Funded studies**<br>Enter a statement with the following details:<br>• Initials of the authors who received each award<br>• Grant numbers awarded to each author<br>• The full name of each funder<br>• URL of each funder website<br>• Did the sponsors or funders play any role in the study design, data collection and analysis, decision to publish, or preparation of the manuscript?<br>• **NO** - Include this sentence at the end of your statement: *The funders had no role in study design, data collection and analysis, decision to publish, or preparation of the manuscript.*<br>• **YES** - Specify the role(s) played.<br><br>* typeset	
**Competing Interests**<br><br>Use the instructions below to enter a competing interest statement for this submission. On behalf of all authors, disclose any competing interests that could be perceived to bias this work—acknowledging all financial support and any other relevant financial or non-financial competing interests.<br><br>This statement is required for submission and **will appear in the published article** if the submission is accepted. Please make sure it is accurate and that any funding sources listed in your Funding Information later in the submission form are also	The authors have declared that no competing interests exist.

declared in your Financial Disclosure statement.<br><br>View published research articles from *PLOS ONE* for specific examples.<br><br>**NO authors have competing interests**<br><br>Enter: *The authors have declared that no competing interests exist.*<br><br>**Authors with competing interests**<br><br>Enter competing interest details beginning with this statement:<br><br>*I have read the journal's policy and the authors of this manuscript have the following competing interests: [insert competing interests here]*<br><br>* typeset	
**Ethics Statement**<br><br>Enter an ethics statement for this submission. This statement is required if the study involved:<br><br>• Human participants<br>• Human specimens or tissue<br>• Vertebrate animals or cephalopods<br>• Vertebrate embryos or tissues<br>• Field research<br><br>Write "N/A" if the submission does not require an ethics statement.<br><br>General guidance is provided below. Consult the submission guidelines for detailed instructions. **Make sure that all information entered here is included in the Methods section of the manuscript.**	Before implementing this study, ethical clearance was sought and obtained from the Institutional Review and Ethics Committee (IREC) of MTRH-Moi University. Approval Number 0003781.

**Format for specific study types**<br><br>**Human Subject Research (involving human participants and/or tissue)**<br>• Give the name of the institutional review board or ethics committee that approved the study<br>• Include the approval number and/or a statement indicating approval of this research<br>• Indicate the form of consent obtained (written/oral) or the reason that consent was not obtained (e.g. the data were analyzed anonymously)<br><br>**Animal Research (involving vertebrate animals, embryos or tissues)**<br>• Provide the name of the Institutional Animal Care and Use Committee (IACUC) or other relevant ethics board that reviewed the study protocol, and indicate whether they approved this research or granted a formal waiver of ethical approval<br>• Include an approval number if one was obtained<br>• If the study involved *non-human primates*, add *additional details* about animal welfare and steps taken to ameliorate suffering<br>• If anesthesia, euthanasia, or any kind of animal sacrifice is part of the study, include briefly which substances and/or methods were applied<br><br>**Field Research**<br><br>Include the following details if this study involves the collection of plant, animal, or other materials from a natural setting:<br>• Field permit number<br>• Name of the institution or relevant body that granted permission	
**Data Availability**<br><br>Authors are required to make all data underlying the findings described fully available, without restriction, and from the time of publication. PLOS allows rare exceptions to address legal and ethical concerns. See the PLOS Data Policy and FAQ for detailed information.	Yes - all data are fully available without restriction

Powered by Editorial Manager® and ProduXion Manager® from Aries Systems Corporation

A Data Availability Statement describing where the data can be found is required at submission. Your answers to this question constitute the Data Availability Statement and **will be published in the article**, if accepted.<br><br>**Important:** Stating 'data available on request from the author' is not sufficient. If your data are only available upon request, select 'No' for the first question and explain your exceptional situation in the text box.<br><br>Do the authors confirm that all data underlying the findings described in their manuscript are fully available without restriction?	
**Describe where the data may be found in full sentences. If you are copying our sample text, replace any instances of XXX with the appropriate details.**<br><br>• If the data are **held or will be held in a public repository**, include URLs, accession numbers or DOIs. If this information will only be available after acceptance, indicate this by ticking the box below. For example: *All XXX files are available from the XXX database (accession number(s) XXX, XXX.).*<br>• If the data are all contained **within the manuscript and/or Supporting Information files**, enter the following: *All relevant data are within the manuscript and its Supporting Information files.*<br>• If neither of these applies but you are able to provide **details of access elsewhere**, with or without limitations, please do so. For example:<br><br>*Data cannot be shared publicly because of [XXX]. Data are available from the XXX Institutional Data Access / Ethics Committee (contact via XXX) for researchers who meet the criteria for access to confidential data.*<br><br>*The data underlying the results presented in the study are available from (include the name of the third party*	All relevant data are within the manuscript and its Supporting Information files.

and contact information or URL).<br>• This text is appropriate if the data are owned by a third party and authors do not have permission to share the data.<br><br>* typeset	
Additional data availability information:	

Powered by Editorial Manager® and ProduXion Manager® from Aries Systems Corporation

Manuscript

Click here to access/download;Manuscript;Dr. Albertine Njie PLoS Manuscript_submitted 04082022.docx

Assessment of effective use of APGAR score in comparison to the gold standard in diagnosis of birth asphyxia at Moi Teaching and Referral Hospital, Kenya.

Njie Albertine Enjema[1], *Winstone Mokaya Nyandiko*[1,2], *Ahoya Phinehas Ademi*[1], *Moutchia Suh Jude*[3]

Author Affiliation:

1. Department of Child health and Pediatrics, College of Health Sciences - Moi University
2. Academic Model Providing Access to Healthcare, Eldoret, Kenya
3. Department of Biostatistics, Epidemiology and Informatics -Perelman School of Medicine at the University of Pennsylvania, Philadelphia, Pennsylvania – USA.

Funding: This study did not receive any funding.

* Corresponding Author:

Njie Albertine Enjema
Department of Child health and Pediatrics,
Moi University College of Health Sciences
P.O. Box 4606-30100, Eldoret-Kenya.
Tel: +254741448909
Email: njieenjema@gmail.com

Running Head: Effectiveness of APGAR score in the diagnosis of birth asphyxia.

ABSTRACT

Background: Birth asphyxia is a consistent key contributor to neonatal morbidity and mortality, notably in sub-Saharan Africa. The APGAR score is a globally used diagnostic tool for birth asphyxia; but there is paucity of data comparing this score with the gold standard (umbilical cord blood pH <7 with neurologic involvement), as well as limited studies assessing how effectively healthcare providers apply the score.

Objective: This study determined how effectively the APGAR score is used in Moi Teaching and Referral Hospital (MTRH) compared to the gold standard in diagnosis of birth asphyxia and identified the healthcare provider factors that affect the effective use of the score.

Methods: Using a quantitative cross-sectional hospital-based design, term babies born in MTRH who weighed ≥2500g were randomly and systematically sampled; and healthcare providers who assign APGAR scores were enrolled via a census. Umbilical cord blood was drawn at birth and at 5minutes for pH analysis. APGAR scores assigned by healthcare providers were recorded. Effective use of the APGAR score was determined by sensitivity, specificity, positive and negative predictive values. At a significance level of 0.05, multiple logistic regression analysis identified independent healthcare provider factors associated with effective use of the APGAR score.

Results: We enrolled 102 babies, and 50 (49%) were females. Among the 64 healthcare providers recruited, 40 (63%) were female and the median age was 34.5years [IQR: 31.0, 37.0]. Assigned APGAR scores had a sensitivity of 71% and specificity of 89%. Babies who were resuscitated, those born via instrumental delivery, and those scored by healthcare providers without access to APGAR scoring charts were significantly more likely to get incorrect APGAR scores.

Conclusion: Assigned APGAR scores had low sensitivity and positive predictive values. Healthcare provider factors independently associated with effective APGAR scoring include instrumental delivery, lack of access to APGAR scoring charts, and neonatal resuscitation.

Keywords: APGAR Score, umbilical cord pH, Birth Asphyxia, Western Kenya.

INTRODUCTION

Perinatal mortality is still a significant public health issue, with its top three aetiologies identified as; prematurity (28%), neonatal infections (26%), and birth asphyxia (23%) [1,2]. Birth asphyxia is a neonatal condition characterized by failure to initiate or sustain breathing at birth, and it has been a consistent major aetiology of neonatal mortality in the last decade [3]. Globally, neonatal mortality accounts for about half of under-five mortality [4]. From 2003-2014, childhood mortality trends in Kenya showed that, among neonatal, infant, and under-5 mortalities, neonatal mortality showed the slowest decrease [5]. Therefore, to decrease the mortality rate of children below five years of age, it is imperative to decrease neonatal mortality [6]. To further curb neonatal mortality, there is a profound need to explore the aetiologies of neonatal mortality such as birth asphyxia, especially in resource-poor settings [7]. Sub-Saharan countries bear the greatest burden of birth asphyxia, and these asphyxia-related deaths are for the most part preventable deaths [8] .

The APGAR score, though widely used for birth asphyxia diagnosis can either overestimate or underestimate asphyxia due to its subjective nature [9]. The combined APGAR score has shown some superiority to the conventional APGAR score in birth asphyxia diagnosis [10,11], and over the years, severely asphyxiated babies have been found to have normal APGAR scores [12,13]. However, in low- and middle- income countries, the conventional APGAR score continues to be solely used for birth asphyxia diagnosis. An APGAR scores of less than <7 depict asphyxia; to ascribe this asphyxia to an intrapartum hypoxic-ischemic event, the American College of Obstetricians and Gynaecologists and American Academy of Paediatrics require an add-on umbilical cord pH value of <7, evidence of neurologic involvement (e.g., seizures, altered tone, coma) and evidence of multiorgan involvement [14]. On the other hand, umbilical cord blood pH has been shown to have better diagnostic value in birth asphyxia [15,16], and when combined with the APGAR score, higher sensitivity in birth asphyxia diagnosis has been observed [17,18].

Birth asphyxia is a major contributor cause of neonatal morbidity and mortality. Globally, it contributes to a quarter of all neonatal deaths. In Kenya, according to the KDHS 2014 report, birth asphyxia is the leading cause of neonatal deaths at 31.6% [5]. It has steadily been identified as one of the major aetiologies of neonatal mortalities in the last decade [3], this creates the need to assess the performance of APGAR score as a diagnostic tool for birth asphyxia in comparison to the gold standard. Failure to optimally diagnose birth asphyxia directly interferes with the timeline of managing neonates, further increasing the risk of neonatal mortality.

Published studies report varying proportion of APGAR score sensitivities and specificities of 62.8-99% and 41-81% respectively [11,19,20]. Consistency in APGAR scoring among healthcare

providers has been shown to vary greatly, with the heart rate component of the score having the least inconsistency. Healthcare provider factors influencing APGAR scoring include among others: years of experience of the healthcare provider, cadre of the healthcare provider, number of staff per delivery, nature of resuscitation done by healthcare provider, and the type of delivery [21–23]. Exploring these factors in this study is pivotal in fostering improved usage of the APGAR score by healthcare providers in identifying neonates with asphyxia.

Improvements in antenatal care, delivery and neonatal resuscitation services have been observed in many countries with developing economies. This has led to the establishment of national intervention programs such as “Help Babies Breathe” in Kenya [24,25]. Yet, birth asphyxia consistently remains a major cause of neonatal mortality, creating the need for effective use of diagnostic tools such as the APGAR score. If the diagnosis of birth asphyxia is not effectively done, it cannot be promptly and adequately managed. Consequently, its short- and long-term morbidity, and mortality persistently occur as reflected by the above-mentioned data. This study therefore aimed to determine how effectively the APGAR score is applied in the labour ward and theatre of a tertiary hospital in Kenya. Furthermore, it assessed healthcare provider factors associated with this effective use of APGAR score. This knowledge will aid in addressing and halting asphyxia-related morbidity and mortality through evidence-based data.

MATERIALS AND METHODS

This was an analytical cross-sectional hospital-based quantitative study carried out in the labour ward, delivery rooms and theatre of Moi Teaching and Referral Hospital (MTRH), Eldoret, between June 2021 and October 2021. The hospital is a level 6 public hospital and is currently the second largest national teaching and referral hospital in Kenya. Its delivery unit, obstetrics theatre and newborn unit (NBU) are located at the Riley Mother and Baby wing of the hospital. An average of 750 deliveries are carried out and approximately 250 babies are admitted to NBU monthly, creating an optimal study setting for birth asphyxia. The researchers randomly and systematically sampled 102 term neonates (37-42weeks gestation) who had a birthweight of at least 2500 grams. To assess healthcare provider factors, 64 healthcare providers who assign APGAR scores to neonates after delivery were purposively sampled. Interviewer-administered semi-structured questionnaires (*Supplementary Material 1*) were used to collect neonatal sociodemographic and clinical characteristics. To collect information on the participant sociodemographic and factors influencing APGAR scoring among healthcare workers, a self-administered questionnaire

(*Supplementary Material 2*) was used. Umbilical cord blood was collected at birth and in the fifth minute of life from the neonates for pH analysis. This was in accordance to the Standard operating procedure (SOP) adapted from the 2015 guidelines of the Research Centre for Women's and Infants' Health (RCWIH) BioBank in Canada for umbilical cord blood collection (*Supplementary Material 3).* APGAR scores in the first, fifth and tenth minutes as routinely given after delivery by healthcare providers were documented; as well as data on neonates who had altered tone and/or clinically apparent seizures within the first 24 hours of life.

Counts and proportions were used to summarise categorical data while median and interquartile range were used to summarise continuous variables. The chi squared test (or Fisher's exact test if any expected cell count was <5) was used to assess significant differences in categorical variables across asphyxia and no asphyxia groups. The Wilcoxon rank sum test was used to test association between predictors and outcomes for continuous variables. Effective APGAR score use was computed as sensitivity, specificity, positive and negative predictive values. Intraclass correlations did not identify the need for multi-level regression models in this study; thus, one-level logistic regression was used at a 95% confidence level to determine independent factors associated with effective APGAR score use, with the dependent variable being incorrect APGAR scores.

RESULTS

This study enrolled 102 neonates, of whom, majority were males 52 (51%). The median [IQR] birth weight and gestational age were 3,010g [IQR: 2,752, 3,375] and 39.0 [IQR: 37.0,40.2] weeks respectively. Most neonates, 65 (63.7%), were born via spontaneous vaginal delivery. Labour was induced in 11 deliveries (10.8%), with the most frequent indication being premature rupture of membranes (5 [45.5%]), seconded by postdate pregnancies (4 [36.4%]). The median [IQR] APGAR score at the 5th minute was 9.0 [IQR: 8.0, 10.0], and 24 (23.5%) neonates were observed to have an APGAR score of <7 at this 5th minute. The median [IQR] cord blood pH at birth was 7.20 [7.09, 7.29] and 7.20 [7.13, 7.28] at the 5th minute. Eleven neonates (10.8%) had clinically apparent seizures, while 17 (16.7%) were noted to have altered tone (Table 1).

Table 1: Neonatal sociodemographic and clinical characteristics

**Characteristic**	**Overall, N = 102**	**No asphyxia, N = 81**	**Asphyxia[1], N = 21**	**p-value[2]**
**Sex of the neonate, n (%)**				**0.04**
Female	50 (49.0)	44 (54.3)	6 (28.6)	
Male	52 (51.0)	37 (45.7)	15 (71.4)	
**Weight (g), median [IQR]**	3,010 [2,752, 3,375]	3,015 [2,795, 3,320]	3,000 [2,620, 3,500]	0.82
**Gestational age (months), median [IQR]**	39.0 [37.0, 40.2]	39.0 [37.0, 40.1]	39.5 [38.0, 41.0]	0.2
**Induction of labour, n (%)**	11 (10.8)	7 (8.6)	4 (19.0)	0.23
[1] **Indication for induction of labour, n (%)**				0.27
Post date	4 (36.4)	3 (42.9)	1 (25.0)	
PROM	5 (45.5)	4 (57.1)	1 (25.0)	
SPET	2 (18.2)	0 (0.0)	2 (50.0)	
**Method of delivery, n (%)**				**0.014**
Caesarean section (CS)	34 (33.3)	28 (34.6)	6 (28.6)	
Instrumental vaginal delivery (IVD)	3 (2.9)	0 (0.0)	3 (14.3)	
Normal vaginal delivery	65 (63.7)	53 (65.4)	12 (57.1)	
**APGAR score at birth**				
Median [IQR]	8.0 [7.3, 9.0]	9.0 [8.0, 9.0]	7.0 [6.0, 7.0]	**<0.001**
APGAR score < 7, n (%)	26 (25.5)	10 (12.3)	16 (76.2)	**<0.001**
**5th minute APGAR score**				

Median [IQR]	9.0 [8.0, 10.0]	9.0 [9.0, 10.0]	7.0 [6.0, 8.0]	**<0.001**
APGAR score < 7, n (%)	24 (23.5)	9 (11.1)	15 (71.4)	**<0.001**
**10th minute APGAR score**				
Median [IQR]	10.0 [9.0, 10.0]	10.0 [10.0, 10.0]	8.0 [8.0, 10.0]	**<0.001**
APGAR score < 7, n (%)	6 (5.9)	2 (2.5)	4 (19.0)	**0.016**
**Cord blood pH at birth, median [IQR]**	7.20 [7.09, 7.29]	7.23 [7.14, 7.32]	7.00 [6.97, 7.00]	**<0.001**
**Cord blood pH at the 5th minute, median [IQR]**	7.20 [7.13, 7.28]	7.21 [7.15, 7.30]	7.12 [7.06, 7.16]	**<0.001**
**Clinically-apparent seizures, n (%)**	11 (10.8)	0 (0.0)	11 (52.4)	**<0.001**
**Altered tone, n (%)**	17 (16.7)	0 (0.0)	17 (81.0)	**<0.001**

[1] *Amongst the 11 babies born post induced labour*
[2] *p values from Wilcoxon rank sum test, Fisher's exact test, and Pearson's Chi-squared test as appropriate*

Among all the 102 neonates enrolled, 21 (21%) had birth asphyxia defined as cord blood pH at birth <7 and presence of clinically apparent seizures and/or an altered tone. The median [IQR] APGAR score was significantly lower amongst neonates with birth asphyxia compared to neonates without birth asphyxia (p <0.001) at birth (7.0 [IQR: 6.0, 7.0] vs. 9.0 [IQR: 8.0, 9.0]), at the 5th minute (7.0 [IQR: 6.0, 8.0] vs. 9.0 [IQR: 9.0, 10.0]), and at the 10th minute (8.0 [IQR: 8.0, 10.0] vs. 10.0 [IQR: 10.0, 10.0]) – Figure 1.

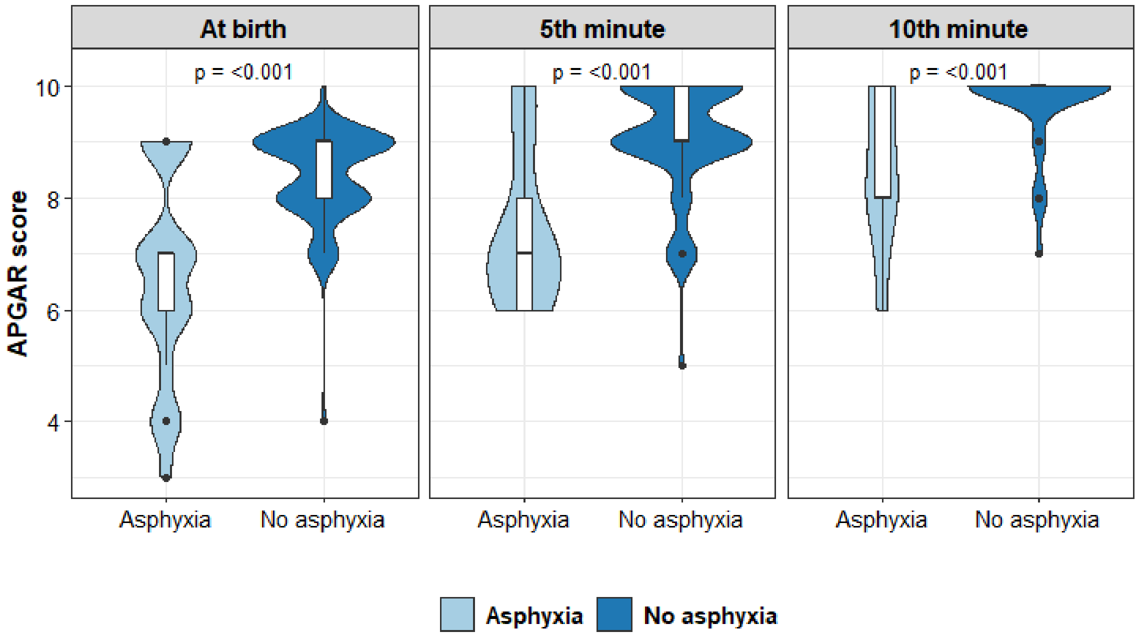

Figure 1: Violin plots showing the distribution of APGAR score at various time points by birth asphyxia status

A total of 64 healthcare workers assigned APGAR scores to the 102 neonates. The median [IQR] age of the healthcare workers was 34.5 [31.0, 37.0] years and 40 (63%) were female. Of these healthcare workers, there were 13 (20%) students, 3 (5%) medical officers, 31 (49%) midwives, 13 (20%) paediatric residents, and 4 (6%) paediatric consultants. Majority (50%) of the healthcare providers had between five- and ten-years working experience with the minimum (2%) observed to have worked for more than 15 years.

Table 2: Socio-demographic characteristics of the healthcare providers who scored the neonates

**Characteristic**	**Total, N = 64**
**Age (years), median [IQR]**	34.5 [31.0, 37.0]
**Sex, n (%)**	
**Female**	40 (62.5%)
**Male**	24 (37.5%)
**Cadre, n (%)**	

**Medical officer**	3 (4.7%)
**Midwife**	31 (48.5%)
**Paediatric consultant**	4 (6.2%)
**Paediatric resident**	13 (20.3%)
**Student**	13 (20.3%)
**[1]Education, n (%)**	
**Diploma**	14 (21.9%)
**Higher National Diploma**	37 (57.8%)
**Bachelor's degree**	12 (18.8%)
**Master's degree**	1 (1.6%)
**Years of work experience, n (%)**	
**<5 years**	20 (31.3%)
**5-10 years**	32 (50.0%)
**>10-15 years**	11 (17.2%)
**>15 years**	1 (1.6%)

[1]Amongst midwives

Healthcare providers agreed to considering the following when assigning APGAR scores: 59 (92%) consider the individual parameters of the score, 45 (70%) seek a second opinion before assigning scores and 47 (73%) routinely consider the second opinions, 57 (89%) consider the need for resuscitation, 54 (84%) consider the nature of the resuscitation, 46 (72%) consider the duration of the resuscitation, 32 (50%) consider the time of the day, and 42 (66%) typically consider the type of delivery. Fifty-one (80%) reported having ready access to the APGAR scoring chart; however, only 24 (38%) felt the need to refer to the APGAR scoring chart before assigning a score to a neonate.

Table 3a is a 2 x 2 table of birth asphyxia as diagnosed by an assigned APGAR score of <7 at the 5th minute versus birth asphyxia as diagnosed by a cord blood pH of <7 at birth with the presence of clinically apparent seizures and/or an altered tone (gold standard).

Table 3a: Diagnosis of birth asphyxia using APGAR score at the 5th minute versus cord pH at birth

		**Cord pH at birth with clinically apparent seizures and/or altered tone**		
		Asphyxia	No asphyxia	**Total**
**APGAR score at the 5th minute**	Asphyxia	15 **(True Positives)**	9 (False Positives)	24
	No asphyxia	6 (False Negatives)	72 **(True Negatives)**	78
	**Total**	21	81	**102**

Of the neonates with birth asphyxia, 15 were truly identified by APGAR scoring at the 5th as having birth asphyxia (sensitivity: 0.71 [95% CI: 0.48, 0.89]), and of the neonates without birth asphyxia, 72 were truly identified by APGAR scoring at the 5th minute as not having birth asphyxia (specificity: 0.89 [95% CI: 0.80, 0.95]). Shown on Table 6 is the effective use of APGAR score.

Table 3b: APGAR scoring at the 5th minute for the diagnosis of birth asphyxia

**Parameter**	**Value (95% CI)**
**Accuracy**	0.85 (0.77, 0.92)
**Sensitivity**	0.71 (0.48, 0.89)
**Specificity**	0.89 (0.80, 0.95)
**Positive predictive value**	0.62 (0.41, 0.81)
**Negative predictive value**	0.92 (0.84, 0.97)
**Positive likelihood ratio**	6.43 (3.28, 12.60)
**Negative likelihood ratio**	0.32 (0.16, 0.63)

Neonates born via instrumental vaginal delivery had 8.83 times higher odds (95% CI: 0.79, 199) of having an incorrect asphyxia classification compared to neonates born via normal vaginal delivery. In resuscitated neonates, there was a 23.83 (6.72, 101.99) times significantly higher odds of an incorrect asphyxia classification; however, the nature of

resuscitation done was not a statistically significant finding. Healthcare practitioners without access to an APGAR scoring chart were significantly more likely (OR: 56.0 [95% CI: 12.9, 322.3]) to incorrectly classify asphyxia compared to healthcare practitioners with access to an APGAR scoring chart. Neonate-related factors (sex, and birth weight) were controlled for in the logistic regression analysis.

Table 4: Factors associated with an incorrect asphyxia classification based on APGAR scoring

Variable	OR (95% CI)	*p* value	[1] aOR (95% CI)	*p* value
**Neonate related factors**				
Sex (Female)	0.89 (0.29, 2.70)	0.84	0.85 (0.27, 2.62)	0.78
Birth weight	0.99 (0.99, 1.00)	0.73	1.00 (0.99, 1.00)	0.88
**Healthcare practitioner related factors**				
Age (in years)	1.06 (0.95, 1.20)	0.35	1.06 (0.95, 1.20)	0.32
Sex (Female)	0.60 (0.20, 1.87)	0.38	0.86 (0.19, 1.81)	0.34
**Cadre**		0.74		0.35
Student	Ref.	-	Ref.	-
Midwife	1.75 (0.38, 12.41)	0.51	2.34 (0.49, 17.08)	0.33
Medical officer	2.75 (0.11, 36.59)	0.45	1.05 (0.34, 223.03)	0.13
Paediatric resident	3.38 (0.58, 26.96)	0.19	6.87 (0.98, 65.03)	0.06
Paediatric consultant	2.75 (0.11, 36.59)	0.45	7.09 (0.25, 124.4)	0.18
**Education level (midwives only)**		0.45		0.49
Diploma	Ref.	-	Ref.	-
Higher National Diploma	0.51 (0.16, 1.72)	0.26	0.53 (0.16, 1.83)	0.30
Bachelor's degree	0.19 (0.01, 1.24)	0.14	0.19 (0.01, 1.34)	0.15
Master's degree	-	-	-	-
**Years of experience**		0.75		0.77
<5 years	Ref.	-	Ref.	-
5-10 years	0.29 (0.01, 1.92)	0.27	0.31 (0.02, 2.2)	0.31
>10-15 years	-	-		
>15 years	0.89 (0.28, 2.96)	0.84	0.99 (0.30, 3.43)	0.98
**Method of delivery**		0.03		**0.04**
Normal vaginal delivery	Ref.	-	Ref.	-
Caesarean section (CS)	0.13 (0.01, 0.73)	0.05	0.12 (0.01, 0.77)	0.06
Instrumental vaginal delivery (IVD)	8.83 (0.79, 199)	0.08	9.87 (0.81, 236)	0.08
**Individual APGAR score parameters**	0.41 (0.02, 2.31)	0.27	0.42 (0.02, 2.45)	0.43

**The need for resuscitation**	23.83 (6.72, 101.99)	<0.001	27.82 (7.29, 134.80)	**<0.001**
**Nature of resuscitation**		0.99		0.98
Suctioning/stimulation	Ref.	-	Ref.	-
Bag/mask ventilation	1.00 (0.07, 13.85)	1.00	0.67 (0.01, 12.4)	0.78
Chest compressions	0.00 (-, -)	0.99		0.99
Intubation	0.00 (-, -)	0.99		0.99
Oxygenation	0.67 (0.04, 10.03)	0.77	0.35 (0.01, 7.22)	0.52
**Lack of access to APGAR score chart**	56.0 (12.9, 322.3)	<0.001	78.9 (15.5, 661.7)	**<0.001**

[1]Odds ratio adjusted for gestational age, sex of the neonate, and the birth weight of the neonate, **one-level logistic regression**

DISCUSSION

This cross-sectional hospital-based study of 102 neonates and 64 healthcare providers, aimed to evaluate the effective use of the APGAR score in comparison to the gold standard, and bring to light what healthcare provider factors are associated with the effective use of the score in our setting.

Effective use of the APGAR score

We estimated a sensitivity of 71% and a specificity of 89%. Our specificity was more comparable to that of a study prospective cohort study conducted among 464 neonates admitted to a tertiary hospital in Iran at 81% [11]. On the other hand, we had a lower sensitivity of 71% compared to the 81% in Iran [11]. Our results therefore show that, the APGAR scores assigned in MTRH need to be improved upon for proper identification of neonates with asphyxia.

We postulate that, our low sensitivity can be explained by a form of ascertainment bias, which is also in line with findings from a review on the APGAR score by the score's founder, Dr. Virginia Apgar. She recommended in this review that, the person carrying out the delivery shouldn't be the one assigning the APGAR score as they could have a vested interest in the neonatal outcome, and also have divided attention in view of the need for continual maternal management [13,26]. The practice for most deliveries in MTRH except caesarean deliveries is that the person carrying out the delivery also assigns the APGAR score to the neonate. This could have therefore resulted in incorrectly high APGAR scores, and true asphyxia being reported (per APGAR score) as no

asphyxia, translating to low sensitivity. On the flip side, the scores observed in our study were better at determining babies who did not have birth asphyxia.

According to a multicentre study that assessed umbilical cord blood parameters in the diagnosis of birth asphyxia, a sensitivity of 99% and a specificity of 41% were obtained [20]. These findings were dissimilar to ours. The difference could be explained by the fact that, in this study, APGAR scores in the first minute were used; while, in our study, we used APGAR scores in the fifth minute as our operational definition for birth asphyxia was adopted from the combined consensus of ACOG/AAP.

We observed a positive predictive value of 0.62 and a negative predictive value of 0.92. Park and associates in their study reported a positive predictive value of 19.4 and a negative predictive value of 96.2 [19]. As clearly shown, our negative predictive values are comparable. On the other hand, they had a much lower positive predictive value when compared with ours. This difference could be explained by two things. Firstly, they applied the APGAR score in their study as a predictive tool for neonatal mortality, while we used it for diagnosis of asphyxia in our study. Secondly, they included extremely low birth weight babies in their study, and we did not.

Healthcare provider factors affecting effective use of APGAR score

In the current study, mode of delivery, need for resuscitation and lack of access to an APGAR scoring chart were significantly associated with the effective use of the APGAR score.

Mode of delivery was significantly associated with incorrect APGAR scores, whereby, babies born via instrumental deliveries were 8 times more likely to have incorrect scores when compared with neonates born via spontaneous vaginal delivery. Additionally, neonates born via caesarean deliveries were slightly less likely to be assigned incorrect scores with an odds ratio of 0.13. Our findings can be attributed to the concept of normalcy by which, deliveries requiring intervention are already viewed as a deviation from the norm - spontaneous vaginal deliveries and have a beforehand expectation of poor neonatal outcome. Therefore, it is possible that this could have negatively influenced how effectively the APGAR score was used in deliveries requiring intervention in our study. This was however more applicable to instrumental deliveries than caesarean births in our study and we think it was so because, we included babies born via elective caesarean sections with healthy outcomes in which case, the assigning of APGAR scores is forthright. Our findings agreed to a previous study which reported that neonates born via normal vaginal deliveries were more likely to be better scored than their counterparts born through other methods of delivery [22].

Babies who underwent resuscitation in our study were 23.83 times more likely to be assigned incorrect APGAR scores. We presuppose that, this finding could be attributed to the conventional APGAR score does not take into account neonatal resuscitation. Additionally, given that the person resuscitating is also the one who scores the neonate, there may have been an element of ascertainment bias whereby, some scores could have been assigned incorrectly to evade responsibility of negative neonatal outcomes. Our findings mirror those reported in Cameroon where a significant variation of APGAR scores assigned to resuscitated versus non-resuscitated babies was noted [27]; and those from the Netherlands, where the authors [28] noted a great variability of the respiratory component of the APGAR score among healthcare providers in the context of resuscitation, especially if a neonate is intubated. Furthermore, in Richmond-Virginia [21], subjective APGAR scoring by healthcare providers in the context of neonatal resuscitation was noted, making the authors recommend the use of other scores such as the Expanded APGAR score or Neonatal Resuscitation and Adaptive score to replace the conventional APGAR score in resuscitated babies. We also highlight this need for alternative scoring methods for resuscitated babies.

Healthcare practitioners without access to an APGAR scoring chart were significantly more likely to incorrectly classify asphyxia compared to healthcare practitioners with access to an APGAR scoring chart. We postulate that, having access to an APGAR scoring chart could have resulted in better utilization of the score in terms of more objective application of the score parameters, as opposed to attempting to apply the score off by heart without the score chart. Our findings were identical to those reported in Nigeria [29], where a similar low-resource setting like the current study was used. Additionally, in Malawi, the authors [30] recommended a review of the guidelines of the use of the APGAR score among healthcare providers.

Conclusion and Recommendations

This study reports low sensitivity and positive predictive values of assigned APGAR scores at 71% and 62% respectively among newborns attending a tertiary hospital in Western Kenya. Newborns who were resuscitated, born by instrumental deliveries, and assigned APGAR scores by healthcare providers without access to the APGAR scoring chart were significantly more likely to get incorrect APGAR scores. With the foregoing, there is need to adopt the gold-standard on birth asphyxia diagnosis into the hospital-based neonatal management guidelines at the tertiary hospital and other resource constrained healthcare settings. In addition, improved APGAR scoring is attainable by augmenting availability of APGAR scoring charts, continuous medical education programs on the score, and on neonatal resuscitation. Additional studies should be conducted to explore the applicability of other neonatal assessment scores for resuscitated neonates.

Author Contribution

Conceptualization: N.A.E; Formal Analysis: M.J.; Resources: N.A.E, W.M.N and A.P.A; Data Curation: N.A.E., and M.J.; Writing – Original Draft: N.A.E.; Writing – Review & Editing: N.A.E, W.M.N and A.P.A; Supervision: W.M.N and A.P.A.

REFERENCES

1. Yelamali BC, Panigatti P, Pol R, Talawar KB, Naik S, Badakali A. Outcome of newborn with birth asphyxia in tertiary care hospital - a retrospective study. 2014; 6. Available: http://files/136/Yelamali et al. - Outcome of newborn with birth asphyxia in tertiary.pdf

2. Lawn JE, Cousens S, Zupan J. 4 million neonatal deaths: When? Where? Why? Lancet. 2005;365: 891–900. doi:10.1016/S0140-6736(05)71048-5

3. Ersdal HL, Mduma E, Svensen E, Perlman J. Birth Asphyxia: A Major Cause of Early Neonatal Mortality in a Tanzanian Rural Hospital. Pediatrics. 2012;129: e1238–e1243. doi:10.1542/peds.2011-3134

4. Arunda M, Emmelin A, Asamoah BO. Effectiveness of antenatal care services in reducing neonatal mortality in Kenya: analysis of national survey data. Glob Health Action. 2017;10: 1328796. doi:10.1080/16549716.2017.1328796

5. KDHS. Kenya Demographic and Health Survey 2014. Cent Bur Stat [Kenya] Kenya Demogr Heal Surv. 2014; iii–372. doi:10.3109/03014460.2013.775344

6. Lambon-Quayefio MP, Owoo NS. Examining the Influence of Antenatal Care Visits and Skilled Delivery on Neonatal Deaths in Ghana. Appl Health Econ Health Policy. 2014;12: 511–522. doi:10.1007/s40258-014-0103-z

7. Halim A, Dewez JE, Biswas A, Rahman F, White S, van den Broek N. When, Where, and Why Are Babies Dying? Neonatal Death Surveillance and Review in Bangladesh. PLoS One. 2016;11. doi:10.1371/journal.pone.0159388

8. Organization WH. Every newborn: an action plan to end preventable deaths. Geneva: World Health Organization; 2014. Available: http://files/55/World Health Organization - 2014 - Every newborn an action plan to end preventable d.pdf

9. Chen Z-L, Liu J. Interpretation of the Experts' Consensus on the criteria for the diagnosis and grading of neonatal asphyxia in China. Transl Pediatr. 2013;2: 59–63. Available: http://tp.amegroups.com/article/view/1844

10. Rüdiger M, Braun N, Aranda J, Aguar M, Bergert R, Bystricka A, et al. Neonatal assessment in the delivery room--Trial to Evaluate a Specified Type of Apgar (TEST-Apgar). BMC Pediatr. 2015;15: 18. doi:10.1186/s12887-015-0334-7

11. Dalili H, Nili F, Sheikh M, Hardani AK, Shariat M, Nayeri F. Comparison of the Four Proposed Apgar Scoring Systems in the Assessment of Birth Asphyxia and Adverse Early Neurologic Outcomes. Weitkamp J-H, editor. PLoS One. 2015;10: e0122116. doi:10.1371/journal.pone.0122116

12. Wayenberg JL, Vermeylen D, Bormans J, Magrez P, Müller MF, Pardou A. Diagnosis of severe birth asphyxia and early prediction of neonatal neurological outcome in term asphyxiated newborns. J Perinat Med. 1994;22: 129–136. doi:10.1515/jpme.1994.22.2.129

13. Sabol BA, Caughey AB. Acidemia in neonates with a 5-minute Apgar score of 7 or greater – What are the outcomes? Am J Obstet Gynecol. 2016;215: 486.e1-486.e6. doi:10.1016/j.ajog.2016.05.035

14. American Academy of Pediatrics AA of. Neonatal Encephalopathy and Neurologic Outcome, Second EditionReport of the American College of Obstetricians and Gynecologists' Task Force on Neonatal Encephalopathy. Pediatrics. 2014;133: e1482–e1488. doi:10.1542/peds.2014-0724

15. Practice AC on O. ACOG Committee Opinion No. 348, November 2006: Umbilical cord blood gas and acid-base analysis. Obstet Gynecol. 2006;108:

1319–1322. doi:10.1097/00006250-200611000-00058

16. Malin GL, Morris RK, Khan KS. Strength of association between umbilical cord pH and perinatal and long term outcomes: systematic review and meta-analysis. BMJ. 2010;340. doi:10.1136/bmj.c1471

17. Gao C, Yuan L, Wang J. [Role of pH value of umbilical artery blood in neonatal asphyxia]. Zhongguo Dang Dai Er Ke Za Zhi. 2009;11: 521–524. Available: http://www.ncbi.nlm.nih.gov/pubmed/19650980

18. YU C, Wang H, Zhao F, Wu F, Zhu H, Qin J. Clinical significance of umbilical artery blood gas on neonatal asphyxia diagnosis--《Journal of Tongji University(Medical Science)》2011年01期. 2011. Available: http://en.cnki.com.cn/Article_en/CJFDTotal-TJIY201101016.htm

19. Park JH, Chang YS, Ahn SY, Sung SI, Park WS. Predicting mortality in extremely low birth weight infants: Comparison between gestational age, birth weight, Apgar score, CRIB II score, initial and lowest serum albumin levels. PLoS One. 2018;13: e0192232. doi:10.1371/journal.pone.0192232

20. Parameters CSG of NUCBG. [Multicenter clinical study on umbilical cord arterial blood gas parameters for diagnosis of neonatal asphyxia]. Zhonghua Er Ke Za Zhi = Chinese J Pediatr. 2010;48: 668–673. Available: http://www.ncbi.nlm.nih.gov/pubmed/21092525

21. Jurdi SR, Jayaram A, Sima AP, Hendricks Muñoz KD. Evaluation of a Comprehensive Delivery Room Neonatal Resuscitation and Adaptation Score (NRAS) Compared to the Apgar Score: A Pilot Study. Glob Pediatr Heal. 2015;2: 2333794X15598293. doi:10.1177/2333794X15598293

22. Kostro M, Jacyna N, Głuszczak-Idziakowska E, Sułek-Kamas K, Jakiel G, Wilińska M. Factors affecting the differentiation of the Apgar score and the biochemical correlation of fetal well-being - a prospective observational clinical study. Dev period Med. 2018;22: 238–246. Available: https://www.researchgate.net/publication/328077284_Factors_affecting_the_differentiation_of_the_Apgar_score_and_the_biochemical_correlation_of_fetal_well-being_-_a_prospective_observational_clinical_study

23. Simon L V, Hashmi MF, Bragg BN. APGAR Score. StatPearls. Treasure Island (FL): StatPearls Publishing; 2020. Available: http://www.ncbi.nlm.nih.gov/books/NBK470569/

24. Bang A, Patel A, Bellad R, Gisore P, Goudar SS, Esamai F, et al. Helping Babies Breathe (HBB) training: What happens to knowledge and skills over time? BMC Pregnancy Childbirth. 2016;16: 364. doi:10.1186/s12884-016-1141-3

25. Rule ARL, Maina E, Cheruiyot D, Mueri P, Simmons JM, Kamath-Rayne BD. Using quality improvement to decrease birth asphyxia rates after “Helping Babies Breathe” training in Kenya. Acta Paediatr (Oslo, Norw 1992). 2017;106: 1666–1673. doi:10.1111/apa.13940

26. Apgar V. The newborn (Apgar) scoring system. Reflections and advice. Pediatr Clin North Am. 1966. doi:10.1016/S0031-3955(16)31874-0

27. Chiabi A, Kago DA, Moyo GK, Obadeyi B. Relevance and Applicability of the Apgar Score in Current Clinical Practice. 2019; 7. Available: http://files/51/Chiabi et al. - 2019 - Relevance and Applicability of the Apgar Score in .pdf

28. Lopriore E, Burk G, Walther F, De Beaufort AJ. Correct use of the Apgar score for resuscitated and intubated newborn babies: Questionnaire study. BMJ. 2004;329: 143–144. doi:10.1136/bmj.38117.665197.F7

29. Mukhtar-Yola M, Audu LI, Olaniyan O, Akinbi HT, Dawodu A, Donovan EF. Decreasing birth asphyxia: utility of statistical process control in a low-resource setting. BMJ Open Qual. 2018;7: e000231. doi:10.1136/bmjoq-2017-000231

30. Chikuse B, Chirwa E, Maluwa A, Malata A, Odland J. Midwives' adherence to guidelines on the management of birth asphyxia in Malawi. 2012;2012. doi:10.4236/ojn.2012.24052

Click here to access/download
Supporting Information
Supplementary Material 1_Neonates 04082022.docx

Supplementary Material 2

Click here to access/download
Supporting Information
Supplementary Material 2_HCW 04082022.docx

Click here to access/download
Supporting Information
Supplementary Material 3_RCWIH_04082022.docx

Click here to access/download
Supporting Information
Dr. Albertine Dataset 06062022.xlsx
