# Supplementary material for: A comparative analysis of APGAR score and the gold standard in the diagnosis of birth asphyxia at a tertiary health facility in Kenya
Source: PLoS One. 2023 May 24;18(5):e0285828. doi: 10.1371/journal.pone.0285828 (PMC10208496; doi:10.1371/journal.pone.0285828)
Supplement: Attachment — Submitted filename: Response to Reviewers.docx [file pone.0285828.s006.docx]

The Journal Editorial Manager

Public Library of Science (PLOS)-One

1160 Battery Street
Koshland Building East, Suite 225
San Francisco, CA 94111,

USA.

Dear Sir/Madam,

**REBUTTAL LETTER TO REVIEWER’S COMMENTS**

I would like to submit corrections for my manuscript entitled "***Assessment of effective use of APGAR score in comparison to the gold standard in diagnosis of birth asphyxia at Moi Teaching and Referral Hospital, Kenya.***"

**Reviewer #1**

*The article is technically and scientifically sound. The only suggested typo identified was the concomitant use of "<" "less than". The article was well written. The style of writing, the language and the soundness of the article is commendable.*

**Response:** Thank you for appreciating the manuscript. The concomitant use of ‘<’ and ‘less than’ has been edited. The start of the sentence on line 78 is now ‘**An APGAR score of <7 depicts asphyxia’**

**Reviewer #2:**

*General comment: Consider employing Professional English Language editing services for this manuscript.*

**Response:** The entire document has been grammatically revised appropriately.

1. **Comment 1**: *Title- Consider modifying the title to ' A comparative analysis of APGAR score and the gold standard in the diagnosis of birth asphyxia at a tertiary health facility in Kenya.*

**Response**: This suggestion was agreeable to the authors, and the title has been edited to the new suggested title “**A comparative analysis of APGAR score and the gold standard in the diagnosis of birth asphyxia at a tertiary health facility in Kenya.**”

2. **Comment 2:** *Short title- Expunge repeated phrase 'compared to'*

**Response:** The repeated phrase ‘compared to’ has been expunged.

3. **Comment 3*:*** *Line 66-69- A synonymous phrase for neonatal mortality like 'neonatal death' can be used.*

**Response:** Neonatal death was used as a synonym to neonatal mortality in these highlighted lines.

4. **Comment 4**: *Line 84- Major cause or contributor*

**Response**: Major contributor was used, and the word “cause” has been deleted from the text. The sentence now reads **‘Birth asphyxia is a major contributor to neonatal morbidity and mortality’.**

5. **Comment 5:** *Line 87 - Consider using a linking word for clarity.*

**Response:** This sentence has been rephrased to ‘**Its consistent unfavorable effect on neonatal mortality in the last decade (18), highlights the need to assess the performance of APGAR score as a diagnostic tool for birth asphyxia in comparison to the gold standard’.**

6. **Comment 6:** *Methods - how were the participants recruited? How did the authors arrive at the total number of participants to be used (sample size)? Consider using a flow chart for these aspects.*

**Response:** A flow chart, alongside a paragraph have been added from lines 120-130 to elaborating as follows ‘**The Karimollah formula for accuracy studies (26) was used to estimate the sample size for this study, using a pre-study estimate of 81% sensitivity and specificity obtained by Dalili and collaborates in their study (11).** **The sampling interval (k) calculated for this study was 2. Therefore, the researchers randomly and systematically sampled 102 term neonates (37-42weeks gestation) who had a birthweight of at least 2500 grams. Neonates with life-threatening malformations were excluded because the APGAR score has been shown to be influenced by congenital malformations (15,27); while, neonates at risk of anaemia/sepsis in the immediate postnatal period were excluded as these could alter umbilical cord blood pH values (28). Details on neonatal recruitment are demonstrated on Fig 1 below.**

7. **Comment 7:** *Results- 64% is not majority, it's about two-thirds*

**Response**: This has been edited as **‘About two-thirds of the neonates, 65 (63.7%), were born via spontaneous vaginal delivery’.**

8. **Comment 8:** *Line 217, 234-240 should be rephrased*

**Response:** Line 217 was expunged as it was highlighted in comment 10 below that, the lines 216-219 have been discussed in the abstract and introduction.

**Response:** Lines 234-240, now lines 256-264 have been edited to read **‘A multicenter study that assessed accuracy of the APGAR score and umbilical cord blood parameters in diagnosing birth asphyxia, reported an APGAR score sensitivity of 99% and a specificity of 41% (20). These findings were dissimilar to ours in that, our sensitivity was lower at 71%, and our specificity was higher than theirs at 89%. The difference could be explained by the fact that in the multicenter study, APGAR scores in the first minute were used; while in our study, we used APGAR scores in the fifth minute in our operational definition for birth asphyxia. The use of fifth-minute APGAR scores in the current study was adopted from the combined consensus of the American College of Obstetricians and Gynecologists/American Academy of Pediatrics (ACOG/AAP)’.**

9. **Comment 9:** *There should be a consistent use of decimal points in the tables.*

**Response:** The tables have been edited to reflect a consistent use of decimal points.

10. **Comment 10:** *Discussion- lines 216-219 have previously been discussed in the abstract and introduction.*

**Response:** These lines were expunged.

11. **Comment 11:** *Line 224-225- This should be in the conclusion and recommendation*

**Response:** These lines were deleted from the discussion, and incorporated into the conclusion and recommendations.

12. **Comment 12:** *References - Most references used are over 10 years old. Aren't there are more recent articles on the subject matter?*

**Response:** The references have been updated as applicable to include more recent studies. However, three old references were not edited due to the following reasons;

- Apgar et al, 1966: pivotal study with guidelines on utilization of the APGAR score by the score’s founder, Dr. Virginia Apgar.
- Wayenberg et al, 1994: one of the pioneer studies highlighting occurrence of birth asphyxia in neonates reported to have normal APGAR scores.
- Zhonghua Er, 2010: lone multicenter study on APGAR score accuracy in a developing economy; it also sheds light on the timeline and knowledge gap on the subject.

Yours faithfully,

Corresponding Author,

**Dr. Njie Albertine.**
