# Supplementary material for: A comparative analysis of APGAR score and the gold standard in the diagnosis of birth asphyxia at a tertiary health facility in Kenya
Source: PLoS One. 2023 May 24;18(5):e0285828. doi: 10.1371/journal.pone.0285828 (PMC10208496; doi:10.1371/journal.pone.0285828)
Supplement: Attachment — Submitted filename: Response to Reviewers 29042023.docx [file pone.0285828.s007.docx]

29^th^ April 2023

The Journal Editorial Manager

Public Library of Science (PLOS)-One

1160 Battery Street
Koshland Building East, Suite 225
San Francisco, CA 94111,

USA.

Dear Sir/Madam,

**REBUTTAL LETTER TO REVIEWER’S COMMENTS**

I would like to submit corrections for my manuscript entitled "***Assessment of effective use of APGAR score in comparison to the gold standard in diagnosis of birth asphyxia at Moi Teaching and Referral Hospital, Kenya.***"

**Reviewer #2: Dear author, consider the following minor corrections.**

**Comment 1: *Line 133: Methods: Rephrase to interviewer-administered, not interviewer-filled.***

**Response to Comments 1:** The methods section (now Line 131) has been rephrased to *“Interviewer-administered semi-structured questionnaires”*.

**Comment 2: *Line 165: Result: 51% is approximately half, not majority. Consider rephrasing that.***

The results section (now Line 163) has been rephrased to *“slightly more than half were male, 52 (51%)”.*

Yours faithfully,

Corresponding Author,

**Dr. Njie Albertine.**
